# The putative tumor suppressor gene *EphA7* is a novel BMI-1 target

**DOI:** 10.18632/oncotarget.11279

**Published:** 2016-08-13

**Authors:** Gaëlle Prost, Sebastian Braun, Falk Hertwig, Marcus Winkler, Lucas Jagemann, Sara Nolbrant, Isabelle V. Leefa, Nils Offen, Kenichi Miharada, Stefan Lang, Isabella Artner, Ulrike A. Nuber

**Affiliations:** ^1^ Lund Strategic Center for Stem Cell Biology, Lund University, 22184 Lund, Sweden; ^2^ Current address: Technical University Darmstadt, 64287 Darmstadt, Germany

**Keywords:** Bmi1, EphA7, neural stem cells, DNA methylation

## Abstract

*Bmi1* was originally identified as a gene that contributes to the development of mouse lymphoma by inhibiting MYC-induced apoptosis through repression of *Ink4a* and *Arf*. It codes for the Polycomb group protein BMI-1 and acts primarily as a transcriptional repressor via chromatin modifications. Although it binds to a large number of genomic regions, the direct BMI-1 target genes described so far do not explain the full spectrum of BMI-1-mediated effects. Here we identify the putative tumor suppressor gene *EphA7* as a novel direct BMI-1 target in neural cells and lymphocytes. *EphA7* silencing has been reported in several different human tumor types including lymphomas, and our data suggest *BMI1* overexpression as a novel mechanism leading to *EphA7* inactivation via H3K27 trimethylation and DNA methylation.

## INTRODUCTION

*Bmi1* (B cell-specific Mo-MLV integration site 1) was identified as a gene cooperating with *Myc* in the generation of B-lymphoid tumors [1, 2]. BMI-1 protein is a component of multiprotein complexes that mediate gene silencing via chromatin modifications [3].

*Bmi1* knockout (*Bmi1*^−/−^) mice are smaller than their wild-type littermates and display neurological, hematopoietic and skeletal abnormalities [4]. *Bmi1* mutant cerebellum is strongly decreased in size and shows a reduced thickness and cellularity of the molecular and granular layer. Thymus, spleen and bone marrow of *Bmi1*^−/−^ mice have profoundly reduced cell counts and posterior transformation defects along the vertebral column are present. At the cellular level, it has been demonstrated that *Bmi1* maintains somatic stem cells: *Bmi1* deficiency leads to impaired self-renewal of hematopoietic, neural, bronchioalveolar and intestinal stem cells and reduced numbers of incisor stem cells [5–10]. Loss of *Bmi1* also results in premature lineage specification of hematopoietic stem cells (HSCs) thereby decreasing their number [11]. The opposite effect, increased self-renewal of hematopoietic and neural stem cells is observed upon *Bmi1* overexpression [12–15]. High BMI-1 levels are present in many hematopoietic and solid tumors and a critical role of *Bmi1* for tumor development and maintenance has been reported [16, 17].

How does *Bmi1* exert its cellular functions? BMI-1 is involved in transcription regulation and is part of repressor complexes PRC1 (Polycomb Repressive Complex 1) and BCOR [3]. Each canonical and non-canonical PRC1 complex contains a distinct type of Polycomb group RING finger protein (such as BMI-1 = PCGF4), a RING1A/B ubiquitin ligase and additional proteins [18]. KDM2B (=FBXL10) recruits non-canonical PRC1 to unmethylated CpG islands and the RING1B component of this complex monoubiquitylates histone H2A on lysine 119 (H2A119ube1) [19–21]. This enzymatic activity is stimulated by BMI-1 [22]. H2A119ube1 deposition leads to the recruitment of Polycomb Repressive Complex 2 (PRC2) which in turn places the repressive H3K27me3 histone mark (trimethylated histone H3 at lysine 27) [23, 24]. Upon binding to H3K27me3, canonical PRC1 can be recruited by CBX proteins.

Although several cell context-dependent BMI-1 effects can be attributed to a number of identified target genes (e.g. *Ink4a/Arf*, [25, 26]; *Cdkn1a [9]*, *Cdkn1c* [27], *Hoxc13* [22], imprinted gene loci [27]; genes involved in TGF-β/BMP and ER stress response pathways [28]) and protein interaction partners (e.g. E4F1 [29], p53 [30]), these do not explain the full spectrum of BMI-1-mediated cell functions.

In this study, we identified the tumor suppressor gene *EphA7* as a novel direct BMI-1 target.

*Bmi1*^−/−^ knockout mice have a reduced number of proliferating cells in a postnatal neural stem cell niche, the LVW. Here we demonstrate that BMI-1 represses *EphA7* in mouse neural stem/progenitor cells and that *EphA7* deletion partially rescues the proliferative defect in the *Bmi1*^−/−^ lateral ventricle wall (LVW). Moreover, we show that elevated BMI-1 levels lead to an increased amount of H3K27me3 and a delayed increase in DNA methylation at the *EphA7* locus. *EPHA7* is inactivated by DNA hypermethylation in several tumor types, and our data suggest that elevated BMI1 levels contribute to this alteration.

## RESULTS

### Identification of novel BMI-1 target genes in neural stem/progenitor cells overexpressing *Bmi1*

**Figure 1:**
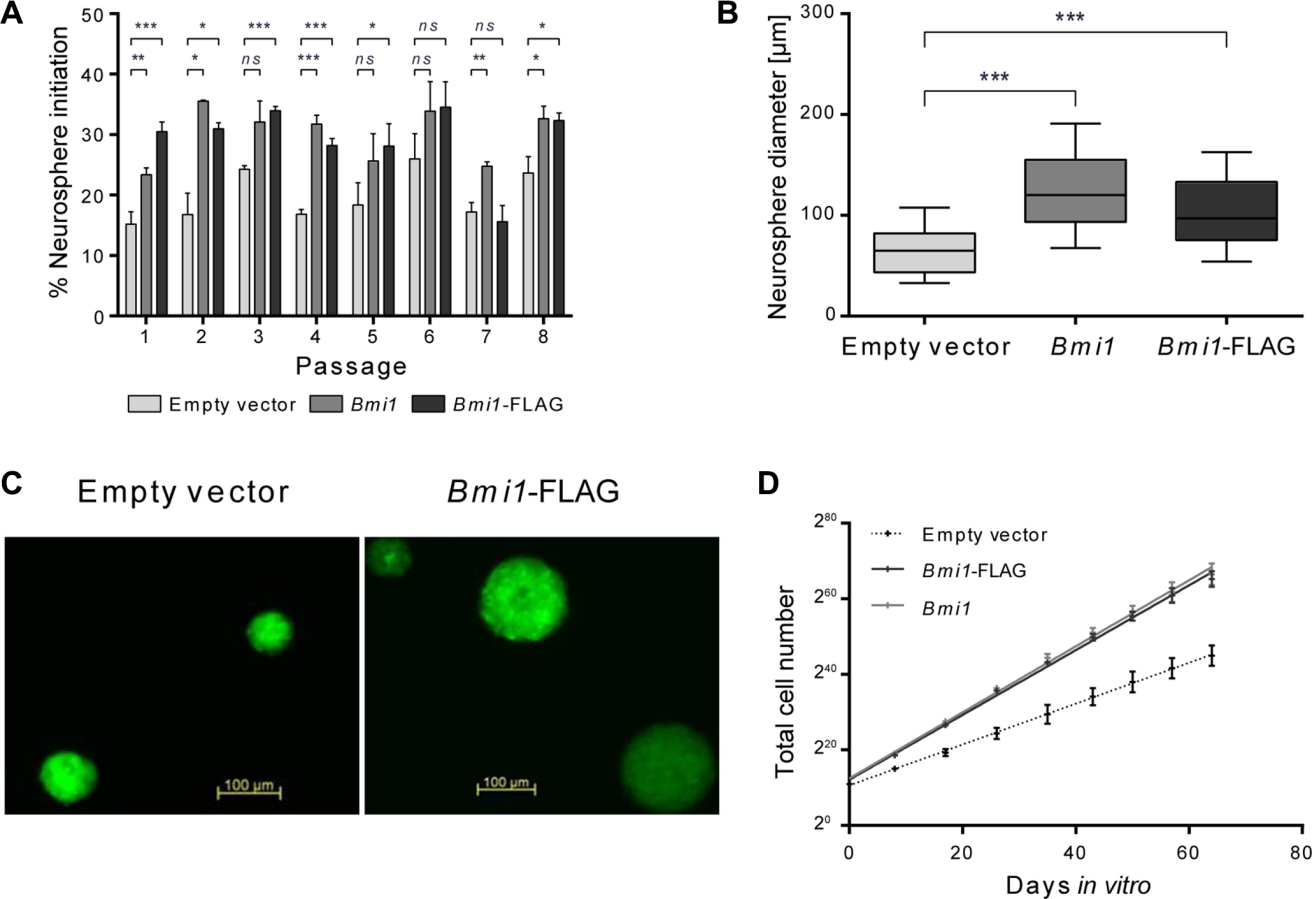
*Bmi1* overexpression increases proliferation and self-renewal of postnatal NSP cells *in vitro* (**A**) Frequency of neurosphere initiation of empty vector, pCMMP-Bmi1 and pCMMP-Bmi1-FLAG transduced cells assessed 7 days post plating for 8 passages (*n* = 3). Error bars represent standard deviations. (**B**) Box plots representing neurosphere diameters determined for empty vector, pCMMP-Bmi1 and pCMMP-Bmi1-FLAG transduced NSP cells at passage 2 (50 spheres were investigated in 3 independent cultures). Whiskers represent the 10–90th percentiles. Results of unpaired *t*-tests in (A) and (B) are marked as follows: *p ≤ 0.05, **p ≤ 0.01, ***p ≤ 0.001, ns: not significant (*p* > 0.05). (**C**) Fluorescent micrographs of empty vector and *Bmi1*-FLAG transduced neurospheres. (**D**) Total cell numbers measured at 7–9 days post plating over 8 passages (*n* = 3). Mean values with standard deviation and linear regression lines are shown. Linear regression analysis showed a significant difference between empty vector and pCMMP-Bmi1 and pCMMP-Bmi1-FLAG transduced NSP cultures (ANCOVA, *p* < 0.0001).

We sought to identify novel BMI-1 target genes in neural stem/progenitor cells which were isolated from the LVW of postnatal mice and grown as neurospheres (NSPs). Test cells were transduced with bicistronic retroviral constructs to overexpress *Bmi1* or FLAG-tagged *Bmi1* (*Bmi1*-FLAG) together with eGFP, and control cells were transduced with an empty vector construct expressing eGFP only. Overexpression of both, flagged and unflagged *Bmi1,* led to the same cellular changes in comparison to empty vector control samples: Increased *in vitro* self-renewal (neurosphere initiation frequency, Figure 1A) and neurosphere size (Figure 1B, 1C). In line with these findings, increased cell numbers were measured in *Bmi1*-overexpressing cultures (Figure 1D).

**Figure 2:**
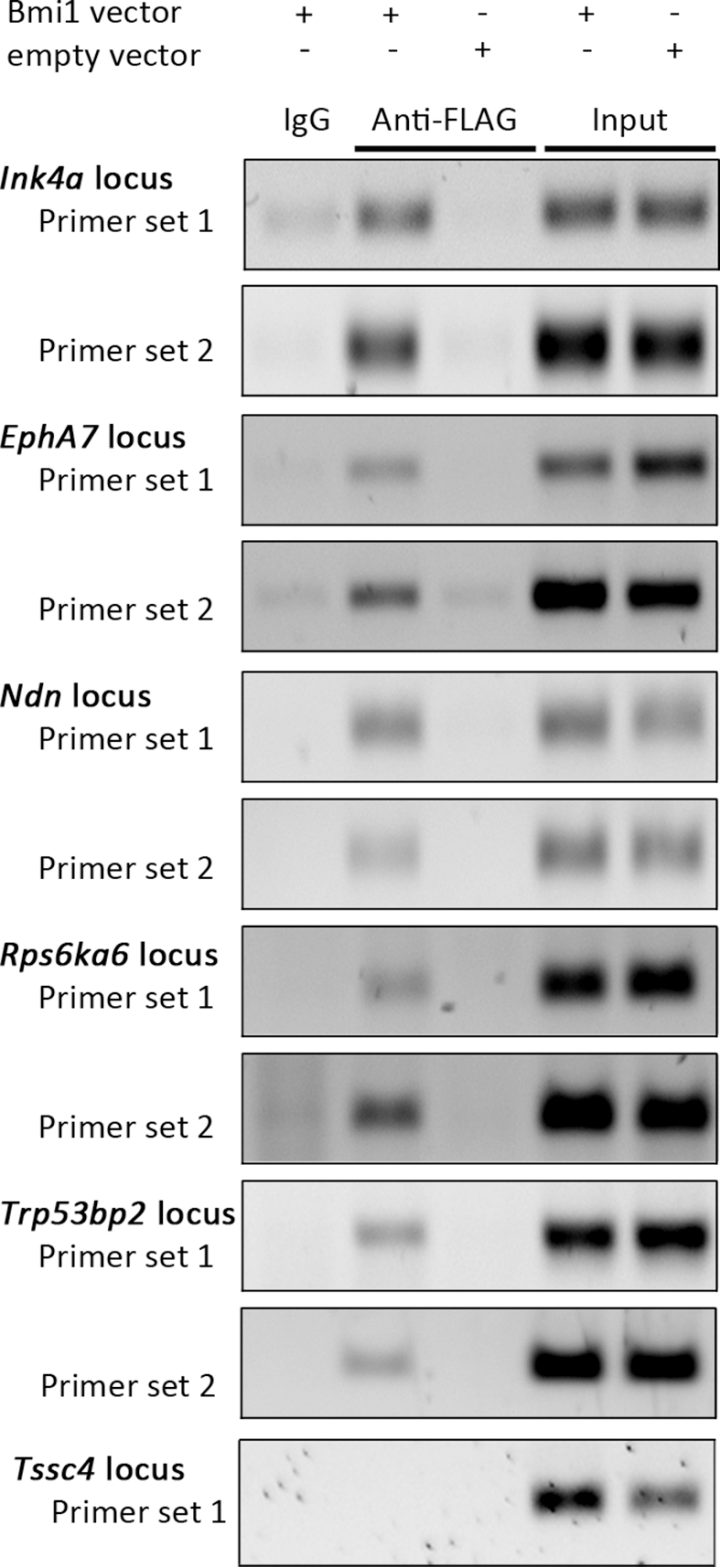
Identification of direct BMI-1 targets by ChIP Representative agarose gel electrophoresis images of PCR-amplified *Ink4a, EphA7*, *Ndn, Rps6ka6, Trp53bp2* and *Tssc4* genomic regions using material from ChIP samples and input controls as template (*n* = 3). ChIP was performed with empty vector (empty) and pCMMP-Bmi1-FLAG-transduced NSP cells, applying the anti-FLAG antibody M2. A matched IgG1 isotype antibody was used as negative control and post-sonication cell lysate served as input control. *Tssc4* PCR results are shown as negative example.

To identify genes which are regulated by BMI-1 in neural stem/progenitor cells we compared the gene expression profile of neurosphere cells overexpressing *Bmi1-FLAG* to control cells using Affymetrix Gene Mouse ST1.0 arrays. Based on the criteria described in Materials and Methods, we obtained 200 differentially expressed sequences which showed a downregulation in *Bmi1*-overexpressing cells, and 100 that were upregulated (Supplementary Information File S1). To identify direct target genes of the transcriptional repressor BMI-1, binding to genomic regions of selected genes that had reduced transcript levels in response to *Bmi1* overexpression was analyzed by chromatin immunoprecipitation (ChIP). Genes with a known or suspected tumor suppressor function were selected. Neurosphere cells overexpressing *Bmi1-FLAG* and an anti-FLAG antibody were used since available BMI-1 antibodies were not suitable for ChIP experiments. Primer pairs spanning the BMI-1-bound *Ink4a* promoter region [26, 31] were used as positive control. A binding of BMI-1 to genomic regions of four novel target genes was detected (Figure 2): *Ndn*, *EphA7*, *Rps6ka6*, and *Trp53bp2*.

### *EphA7* variant transcripts are decreased in *Bmi1*-overexpressing and increased in *Bmi1^−/−^* cells

**Figure 3:**
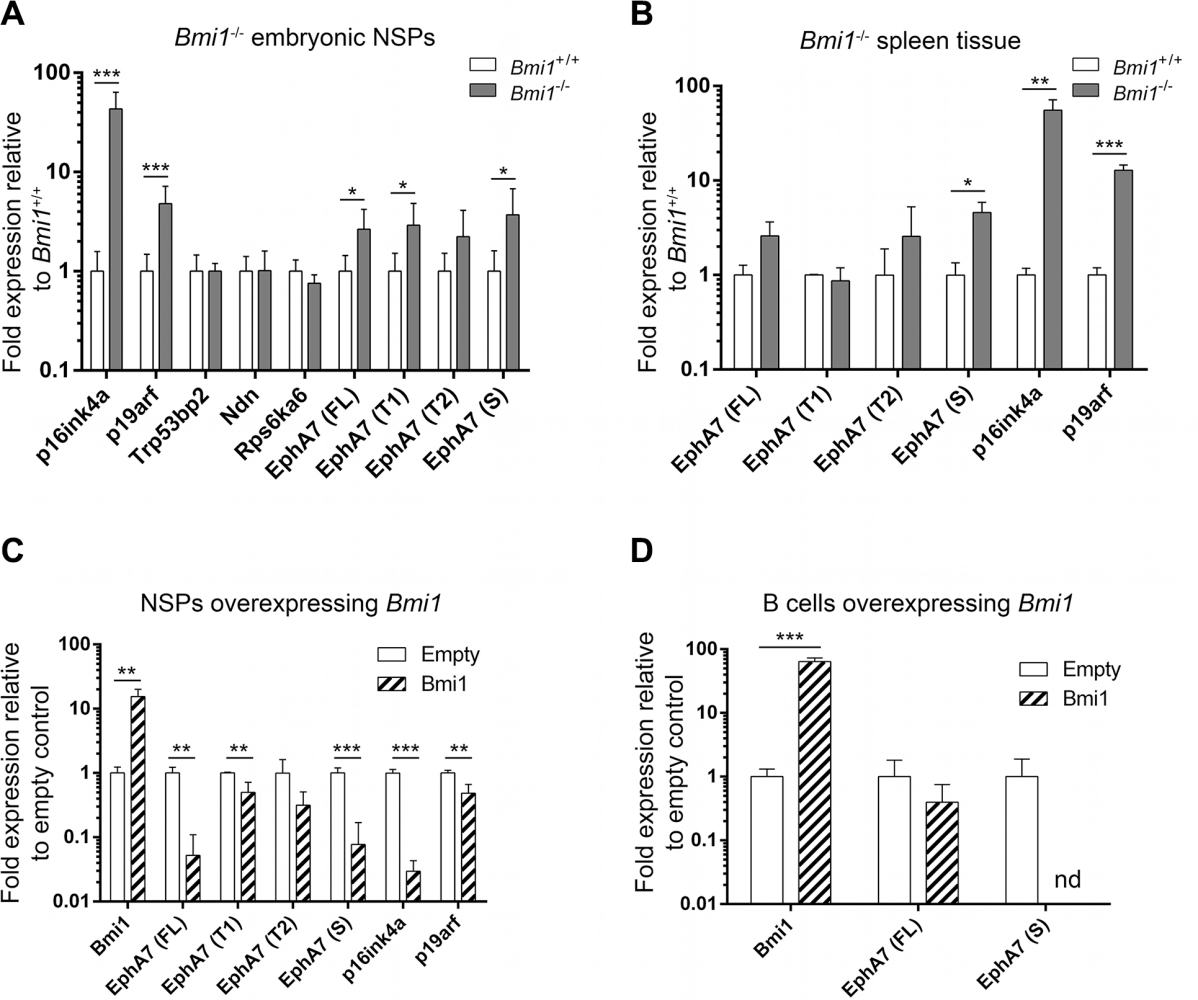
Derepression of *EphA7* isoforms in *Bmi1^−^*^/−^ cells and downregulation in *Bmi1*-overexpressing cells Gene expression levels determined by qPCR analyses are shown. *p16ink4a* and *p19arf* served as positive controls. (**A**) Upregulation of candidate BMI-1 target genes in embryonic day E14.5 neurosphere (NSP) cells isolated from *Bmi1^−/−^* animals in comparison to cells isolated from *Bmi1^+/+^* animals (*n* = 8). Note the increased transcript levels of the full length (FL), truncated (T1) and secreted (S) *EphA7* isoforms (**B**) Upregulation of *EphA7* secreted isoform in postnatal spleen tissue from adult *Bmi1^−/−^* animals in comparison to tissue from *Bmi1^+/+^* mice (*n* = 3). Downregulation of *EphA7* isoform transcripts in postnatal neurosphere cells (**C**) and in postnatal spleen B cells (**D**) in *Bmi1*-overexpressing cells in comparison to empty vector control cells (*n* = 3). Note that upon *Bmi1* overexpression in spleen B cells, transcripts of the secreted *EphA7* isoform were not detectable (nd) anymore. Significant results of unpaired *t*-tests are marked as follows: *p ≤ 0.05, **p ≤ 0.01, ***p ≤ 0.001. Mean values with standard deviation are shown.

We next wanted to know if these novel BMI-1 target genes, which were downregulated upon *Bmi1* overexpression, are conversely derepressed in the absence of *Bmi1*. The expression of the four genes was therefore analyzed by quantitative RT-PCR (qRT-PCR) in neural stem/progenitor cells isolated from *Bmi1^−/−^* and *Bmi1^+/+^* (wild-type) mice. *Bmi1^−/−^* mice frequently die shortly after birth [4] and the growth of adult *Bmi1^−/−^* neurospheres is strongly impaired, thus tissue from embryonic stage (E)14.5 wild-type and mutant animals was used for these experiments. Only *EphA7* was significantly upregulated in *Bmi1^−/−^* embryonic neurospheres while expression of other candidate genes was not affected by loss of *Bmi1* (Figure 3A). In addition to studying full length (FL) *EphA7* transcripts, we investigated alternatively spliced truncated *EphA7* variants (Figure 3A, 3B) since they function differently from FL *EphA7* (see discussion below). *EphA7* T1 and T2 represent truncated *EphA7* mRNAs which lack the intracellular domain [32], and the S variant lacks both the intracellular and transmembrane domain [33], producing a secreted EphA7 protein.

In addition to abnormalities of the nervous system, *Bmi1^−/−^* mice have a hypoplastic bone marrow and severely reduced thymus and spleen [4]. Since EphA/ephrin-A family members are expressed and function in hematopoietic cells and EphA7-S acts as a tumor suppressor in B cells [33, 34], we investigated if *EphA7* is also upregulated in *Bmi1*-deficient cells of mouse spleen. Increased transcript levels of the secreted *EphA7* variant were detected in *Bmi1^−/−^* spleen tissue (Figure 3B).

Our microarray data on *Bmi1*-overexpressing neurospheres did not allow to distinguish the expression of different EphA7 variants and we therefore analyzed the transcript levels of these variants by qRT-PCR. *Bmi1-*FLAG was overexpressed in postnatal neurospheres and spleen B cells. In neurosphere cells harvested two weeks after transduction with *Bmi1*-overexpressing constructs, FL, T1 and S *EphA7* variants were downregulated (Figure 3C); in spleen B cells transcript levels of secreted *EphA7* were lowered below detectable levels in *Bmi1*-overexpressing cells compared to empty vector control cells (Figure 3D). Due to the scarcity of material and the fact that the truncated variants were not significantly upregulated in spleen tissue, we did not investigate the T1 and T2 variants in spleen B cells.

### Deletion of *EphA7* partially rescues the proliferation deficiency in the LVW of *Bmi1^−/−^* mice

Two prominent neural abnormalities in *Bmi1^−/−^* mice are neural stem cell dysfunction and morphological alterations of the cerebellum. Postnatal *Bmi1^−/−^* neural stem cells are severely impaired in their *in vitro* self-renewal capacity determined by neurosphere cultures, and the diameter of neurospheres is significantly smaller compared to neurospheres derived from wild-type animals [5]. The cerebellum of *Bmi1^−/−^* mice is strongly decreased in size and has a reduced thickness and cellularity of the molecular and granular layer [4].

**Figure 4:**
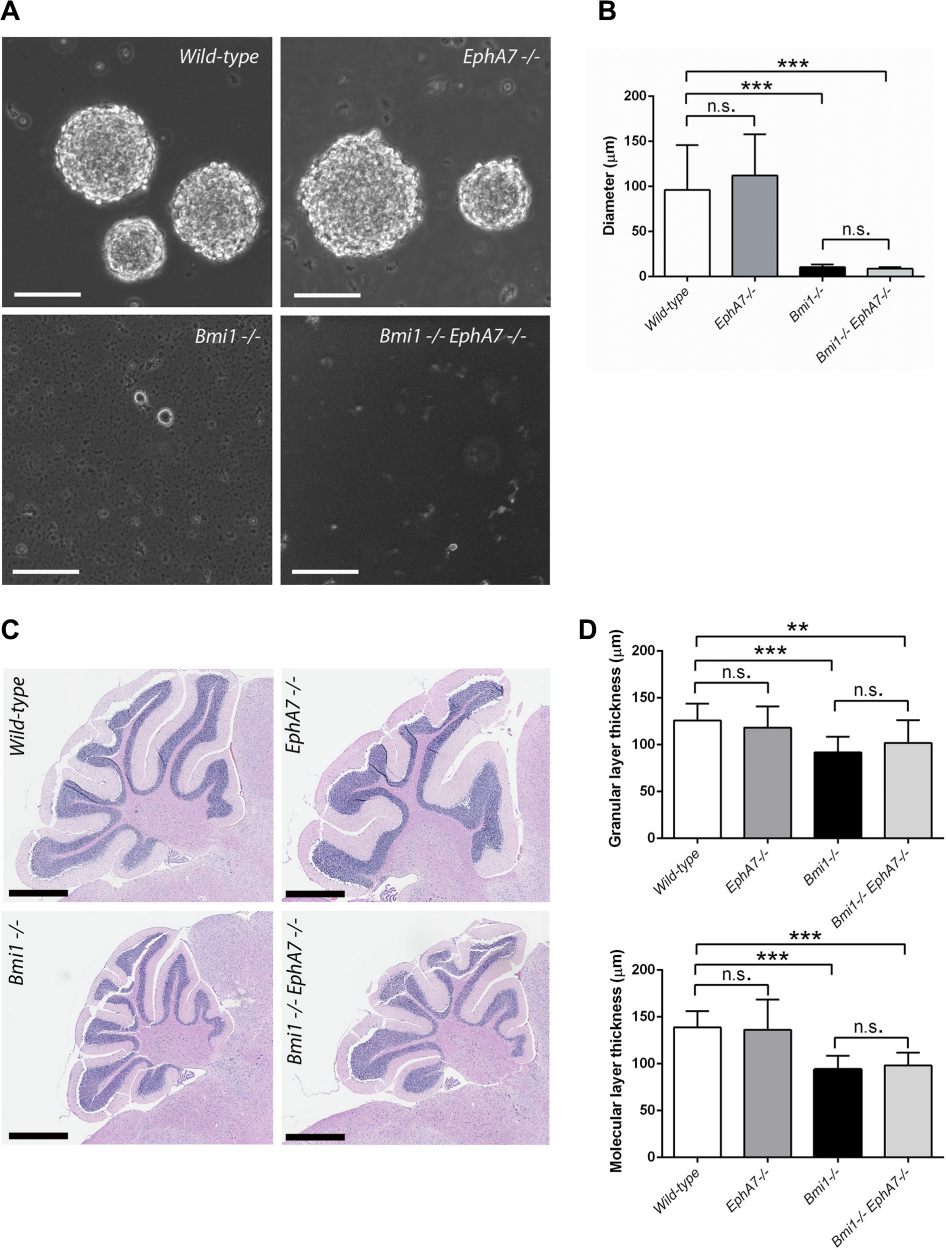
*EphA7* deletion does not rescue postnatal neurosphere and cerebellar defects in *Bmi1*^−/−^ mice (**A**) Neural stem/progenitor cells grown as neurospheres (passage 1). Scale bars 100 μm. (**B**) Comparison of sphere diameters (*n* = 10–13). Mean values with standard deviation are shown. In *Bmi1^−/−^* and *Bmi1^−/−^/EphA7^−/−^* cultures, most of the cells were single cells and did not form aggregates. (**C**) Sagittal sections of paraffin-embedded cerebelli stained with hematoxylin-eosin. Scale bars 800 μm. Cells and tissue were isolated from six week-old mice. (**D**) Granular and molecular layer thickness, mean values with standard deviation. 3 mice per genotype, 3–6 measurements per mouse. Significant results of unpaired *t*-tests are marked as follows: n.s. not significant (*p* > 0.05), **p ≤ 0.01, ***p ≤ 0.001.

To determine if derepression of *EphA7* contributes to the cerebellar and neural stem cell phenotypes in *Bmi1^−/−^* mice, double knockout (*Bmi1^−/−^EphA7^−/−^*) mice were generated by crossing heterozygous (*Bmi1*^+/*−*^) animals with *EphA7^−/−^* mice. The latter carry a null mutation in the *EphA7* gene which results in a complete absence of *EphA7* expression [35]. Neurospheres isolated from both 6 week-old *Bmi1^−/−^EphA7^−/−^* and from *Bmi1^−/−^* single knockout animals were much smaller than those from wild-type and single knockout *EphA7^−/−^* mice (Figure 4A, 4B) and we did not succeed in passaging the *Bmi1^−/−^EphA7^−/−^* and *Bmi1^−/−^* neurospheres more than once. As in *Bmi1^−/−^* mice, the cerebelli from 6 week-old *Bmi1^−/−^EphA7^−/−^* animals were smaller compared to wild-type mice and had a reduced granular and molecular layer thickness (Figure 4C, 4D). In terms of these parameters, no significant difference between *Bmi1^−/−^EphA7^−/−^* and *Bmi1^−/−^* mice was detected. From these findings we conclude that the defects of the *in vitro* potential of postnatal neural stem/progenitor cells lacking *Bmi1* and the cerebellar abnormalities in postnatal *Bmi1^−/−^* mice cannot be rescued by the deletion of *EphA7*.

**Figure 5:**
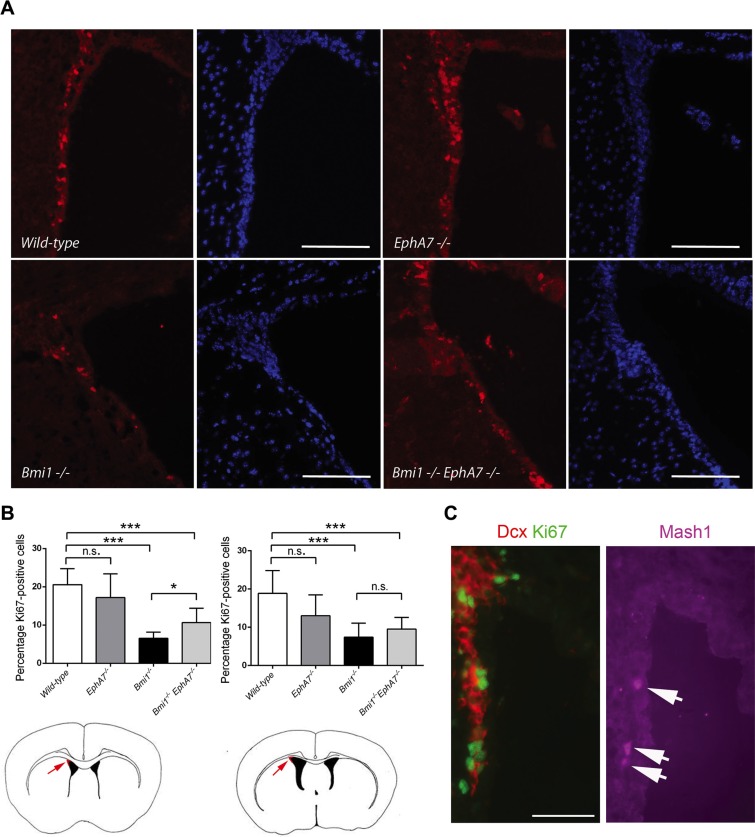
Deletion of *EphA7* increases the number of proliferating cells in the LVW dorsolateral corner of *Bmi1*^−/−^ mice (**A**) Coronal anterior brain sections of 6–7 week-old mice stained with an antibody against Ki67 (red) and DAPI (blue). Scale bars: 100 μm. (**B**) Number of Ki67-positive nuclei per DAPI-positive nuclei shown as percentages (Wild-type: *n* = 10 ventricles, *EphA7^−/−^ n* = 10, *Bmi1*^−/−^ *n* = 8, double knockout *n* = 6). Results of unpaired *t*-tests are marked as follows: *p ≤ 0.05, ***p ≤ 0.001, ns: not significant (*p* > 0.05). Mean values with standard deviation are shown. Cartoons depict anterior (left hand side) and posterior (right hand side) forebrain sections. Arrows point to the dorsolateral corner (red). (**C**) Coronal anterior brain section of a 6 week-old wildtype animal stained with antibodies against Ki67 (green), the neuroblast marker Dcx (red), and the neural progenitor cell marker Mash1 (purple). Arrows point to Mash1-positive cells. Scale bar: 50 μm.

EphA7 is an ephrin receptor and forward and reverse signaling through these receptors depends on the cellular context and the presence of different ligands [36, 37]. Since the microenvironment present in the neural stem/progenitor cell niche of the LVW is not preserved under neurosphere culture conditions, we also analyzed tissue sections of wild-type, *Bmi1^−/−^*, *EphA7^−/−^,* and *Bmi1^−/−^EphA7^−/−^* mice. A lower number of proliferating neural stem/progenitor cells has previously been reported in the dorsolateral corner of the LVW in *Bmi1^−/−^* mice [38]. We counted the number of all (DAPI-positive) and of proliferating (Ki67-positive) cells in the dorsolateral corner of the LVW in anterior and posterior tissue sections and calculated the percentage of Ki67-positive cells (Figure 5A, 5B). A significantly lower percentage of proliferating cells was found in anterior and posterior sections when comparing *Bmi1^−/−^* with wild-type animals (Figure 5B). Moreover, in anterior sections, the percentage of proliferating cells in the dorsolateral corner was significantly higher in *Bmi1^−/−^EphA7^−/−^* compared with *Bmi1^−/−^* animals (Figure 5B). The majority of Ki67-positive proliferating cells in mouse brains of all four genotypes were Dcx-positive neuroblasts; very few Ki67-positive cells were co-stained with the neural progenitor cell marker Mash1 (see Figure 5C and data not shown). From these data we conclude that the absence of *EphA7* in *Bmi1^−/−^* mice increases the percentage of proliferating neuroblasts and neural progenitor cells in the dorsolateral corner of the anterior LVW of *in vivo*, albeit not to the level present in wild-type animals.

### Deletion of *EphA7* does not rescue gross hematopoietic abnormalities in *Bmi1^−/−^* mice

*Bmi1*^−/−^ animals are characterized by a diminished spleen and thymus size and reduced cellularity of the spleen, thymus, and bone marrow [4]. We found a lower spleen and thymus weight and reduced cellularity of spleen, thymus, and bone marrow in *Bmi1^−/−^* compared with wild-type animals. These deficiencies were not significantly improved in *Bmi1^−/−^EphA7^−/−^* animals (Supplementary Information Figure S1) arguing that the BMI-1-*EphA7* axis does not contribute to the consequences of *Bmi1* loss of function in these organs.

### *Bmi1* overexpression increases the DNA methylation of an *EphA7* promoter-associated CpG island

**Figure 6:**
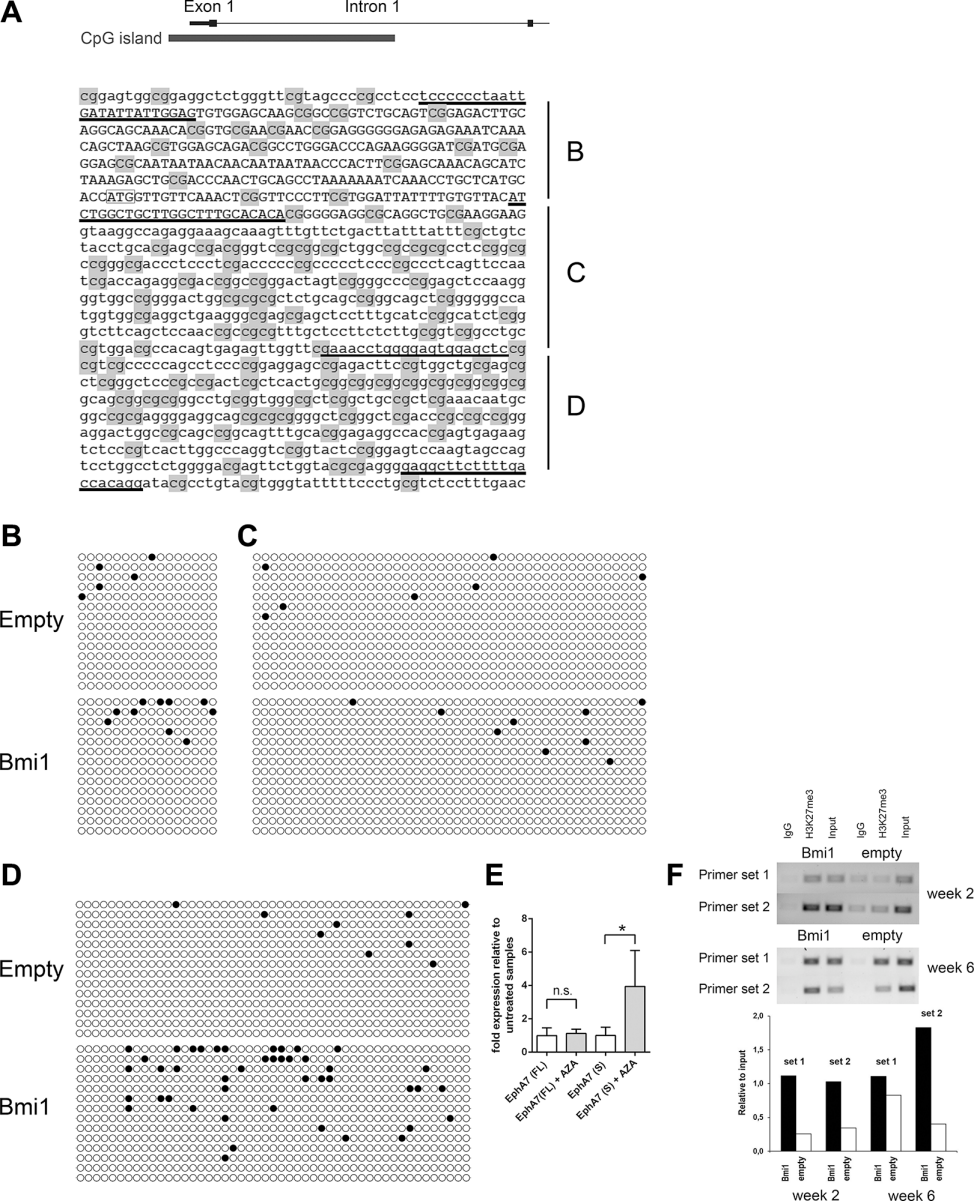
Overexpression of *Bmi1* leads to increased DNA methylation of a genomic sequence within intron 1 of the *EphA7* locus (**A**) Mouse *EphA7* genomic sequence including exon 1 (upper case) and the beginning of intron 1 (lower case). The start codon is marked by a box. Relative position of the CpG island is depicted on top. Underlined sequences indicate primers used to amplify three regions (**B**–**D**). CpGs are highlighted in grey. (B–D) Bisulfite sequence analysis of the three regions. Each row represents the sequence of an individual sequenced clone (open circle: unmethylated CpG site, filled circle: methylated CpG site). Clones from three independent biological samples of *Bmi1-* overexpressing and of empty control neurospheres cultured for 6 weeks after transduction were analyzed. The frequency of methylated CpGs was calculated for each clone and compared between *Bmi1*-overexpressing and empty control samples for each of the three regions. *p*-values of unpaired *t*-tests: region B *p* = 0.3351, region C *p* = 0.5611, region D *p* = 0.0029. (**E**) qRT-PCR of *Bmi1*-overexpressing NSPs cultured for 6 weeks after transduction shows an up-regulation of *EphA7* secreted isoform upon AZA treatment. Results of unpaired *t*-tests (*n* = 3) are marked as follows: *p ≤ 0.05, ns: not significant (*p* > 0.05). (**F**) Agarose gel electrophoresis images of PCR-amplified *EphA7* genomic regions (primer sets 1 and 2) using material from ChIP and input control samples as template. ChIP was performed with empty vector (empty) and pCMMP-Bmi1-FLAG-transduced NSP cells 2 and 6 weeks after transduction, applying an anti-H3K27me3 antibody. A matched IgG1 isotype antibody was used as negative control. Post-sonication cell lysate served as input control. Note the higher relative levels of bound H3K27me3 (H3K27me3 relative to input control) in *Bmi1*-overexpressing cells compared with empty control cells. The primer set 1 region is located 925 bp upstream of the *EphA7* translation start site, the primer set 2 region is 341 bp upstream of this site.

*EPHA7* expression is low to absent in a large fraction of certain human hematological malignancies: germinal center B-cell Non-Hodgkin lymphomas [33], follicular lymphomas [34], and T cell lymphomas [39]. Moreover, reduced to absent *EPHA7* expression has also been found in human colorectal cancer [40]. The reduction of *EPHA7* gene expression in these tumor types can be explained by increased genomic methylation and genomic deletion of this locus with the former being a frequent event. Hypermethylation of *EPHA7* without a corresponding analysis of gene expression levels has also been reported for human prostate cancer tissues and many human acute lymphoblastic leukemia lines and primary samples [41, 42]. Oricchio *et al.* showed that the secreted form of EPHA7 can act as a tumor suppressor in human follicular lymphoma [34]. Its tumor suppressor function is mediated by dominant-negative inhibition of EPHA2 dimerization and activation, thus blocking oncogenic EPHA2 signaling. The cause of *EPHA7* silencing by DNA methylation in tumors has not been determined to date. We hypothesized that *BMI-1* overexpression leads to a downregulation of *EPHA7* expression in human tumors and that this event might then contribute to DNA methylation of the *EPHA7* gene. To test this, the DNA methylation of three regions around the *EphA7* transcriptional start site was analyzed by bisulfite-sequencing in *Bmi1*-overexpressing and empty control neurospheres (Figure 6).

Two weeks after the transduction of neurosphere cells, both empty control and *Bmi1*-overexpressing cells- the latter downregulating *EphA7* (Figure 3C) - contained extremely few methylated cytosines within a promoter-associated CpG island (data not shown). However, in cells analyzed at a later time point - 6 weeks after viral transduction - DNA methylation levels within intron 1 of the *EphA7* promoter-associated CpG island were higher in *Bmi1*-overexpressing cells and significantly different in comparison to empty control cells (Figure 6D, referring to segment “D” in Figure 6A). Addition of the demethylating agent AZA to cells 6 weeks after viral transduction led to an up-regulation of the secreted *EphA7* variant (Figure 6E), indicating that the DNA methylation level present at this time point reduces *EphA7* gene expression.

Interestingly, ChIP experiments revealed increased H3K27me3 levels at the *EphA7* locus at 2 weeks and 6 weeks after transduction, indicating that this repressive mark which is known to be added by the PRC2 complex is present already before the onset of increased DNA methylation (Figure 6F).

Since we found BMI-1 to repress *EphA7* in neural cells and lymphocytes and given that high BMI1 expression levels have been reported in two brain tumor types (medulloblastoma and glioblastoma, [43, 44]) and that the secreted form of EPHA7 functions as tumor suppressor in human follicular lymphoma [34], we asked if high BMI-1 levels might be a cause for the silencing of *EPHA7* in these tumor types. To address this question, the correlation between *BMI1* and *EPHA7* expression levels in human follicular lymphoma, medulloblastoma and glioblastoma samples was calculated (see Materials and Methods for the Affymetrix gene expression datasets used). Spearman's rank correlation coefficients were calculated for gene expression levels of *EPHA7* in comparison to 20 human genes encoding components of mammalian PRC1 and PRC2. These analyses revealed a negative correlation between *BMI1* and *EPHA7* expression levels for the human follicular lymphoma and medulloblastoma datasets, but not for the glioblastoma datasets (Supplementary Information File S2, and data not shown). Taken together, our results support the idea that silencing of *EPHA7* in human follicular lymphoma and medulloblastoma might occur upon *BMI1* overexpression.

## DISCUSSION

In this study we have established *EphA7* as a direct BMI-1 target, which is downregulated upon *Bmi1* overexpression and derepressed in the absence of BMI-1. Eph tyrosine kinase receptors mediate positive and negative effects on cell survival, adhesion, migration, and cell growth depending on the presence of membrane-bound ephrin ligands and co-receptors. Signaling of ephrin ligands/Eph receptors can occur bidirectionally – forward signaling by Eph receptors into the receptor-carrying cell and reverse signaling into the ligand-presenting cell. Truncated EphA7 T1, which lacks the intracellular domain, but retains the transmembrane domain [32], and the secreted variant S, which is devoid of both the intracellular and transmembrane domain [33], block signaling activities of full length EphA receptors [34, 45]. Our qRT-PCR data of *Bmi1*-overexpressing and knockout cells show that BMI-1 can in principle inhibit the expression of full length *EphA7*, the truncated variant T1 and the secreted variant S. Thus, consequences of BMI-1 repressive effects depend on the repertoire of expressed *EphA7* variants in specific cell types. In normal B cells, EphA7 S acts as a tumor suppressor by dominant-negative inhibition of dimerization and activation of oncogenic EPHA2 and our data suggest that *BMI1* overexpression may contribute to the development of tumors such as B-cell derived follicular lymphomas by repressing *EPHA7 S*. Despite the upregulation of *EphA7* variants in *Bmi1^−/−^* cells and tissues, no attenuation of the altered spleen, thymus and bone marrow parameters and the abnormal cerebellar anatomy was observed in *Bmi1^−/−^/EphA7^−/−^* mice. These results indicate that BMI-1 acts through other target genes in these organs. We did however find that deletion of *EphA7* leads to a partial rescue of the reduced cell proliferation in the dorsolateral corner of the LVW in *Bmi1^−/−^* mice. In the postnatal mouse brain, a large number of neural stem cells, specialized types of glia cells, reside in the wall of the lateral ventricles [46]. These cells self-renew and turn into neural progenitor cells, also called transit amplifying C cells, which generate neuroblasts that leave the LVW and migrate along the rostral migratory stream to the olfactory bulb where they differentiate into interneurons. Besides neural stem cells and their progeny, other cell types such as ependymal cells make up the specialized stem cell niche at the LVW. The dorsolateral corner of this region contains the highest number of proliferating cells [47]. Holmberg *et al.* found that ependymal cells and neural stem cells in the adult murine LVW express *EphA7* and that the progeny of neural stem cells in this region, Mash1-positive neural progenitor cells and PSA-NCAM-positive neuroblasts, express the ligand ephrin-A2 [48]. They furthermore showed that this ligand/receptor system acts to negatively regulate neural progenitor proliferation by ephrin-A2 reverse signaling. Our experiments revealed that BMI-1 serves to maintain proliferation in this region and that part of this effect is due to *EphA7* repression. Considering the findings by Holmberg *et al*., this BMI-1-mediated effect could be explained by reduced EphA7 levels leading to an attenuation of the anti-proliferative EphA7/ephrin-A2 reverse signaling in neural progenitor cells. The expression of different *EphA7* variants in adult neural stem/progenitor cells cultured as neurospheres, two of which (T1 and S) have been shown to block full length EphA7 signaling activity, may implicate that multiple mechanisms are present to fine tune the activity of this neural stem cell/progenitor signaling system. Since we did not detect a complete rescue of proliferating cell numbers in *Bmi1^−/−^/EphA7^−/−^* animals, we conclude that other BMI-1 target genes also contribute to this defect. Noticeable differences have been found between *in vivo* and *in vitro* obtained results on BMI-1 functions in neural stem/progenitor cells. The positive effect on neural stem/progenitor self-renewal in cell culture can largely be attributed to the repression of *p16Ink4a* and *p19Arf*, genes which are upregulated in cultured cells [49, 50], but are not expressed in the fetal and young adult brain *in vivo* ([14] and references therein). The fact that in our study, deletion of both *EphA7* and *Bmi1* did not lead to an increased cell number (neurosphere diameter) or neurosphere self-renewal compared to single *Bmi1* knockout *in vitro,* is very likely due to the large effect increased Ink4a/Arf levels have on these cultured cells. In addition, loss of the LVW stem cell niche architecture under cell culture conditions may contribute to the different *in vitro* and *in vivo* results.

Hypermethylation of the *EphA7* gene in human and murine tumors has been detected at CpG islands around the transcriptional start site [33, 34, 39–42] and is associated with a downregulation of *EphA7* transcripts. Our bisulfite sequencing and AZA treatment data suggest that a delayed DNA methylation process at an *EphA7* promoter-associated CpG island can be triggered by *Bmi1* overexpression and that this has an impact on *EphA7* gene expression levels. This late effect occurs after the acute *EphA7* repression that is immediately detectable upon *Bmi1* overexpression and that is accompanied by the presence of the repressive histone modification H3K27me3. CpG islands are associated with approximately 70% of annotated vertebrate gene promoters and are generally DNA methylation-free. In tumor cells however, many promoter CpG islands are hypermethylated and this is associated with chromatin compaction and stable long-term silencing of gene expression [51, 52]. DNA methylation is mediated by DNA methyltransferases (DNMTs), however it remains unclear how this activity is targeted to specific genomic sites [53].

How could elevated levels of the PRC1 component BMI-1 initiate DNA methylation? Several studies provide evidence for a connection between PRC binding/activity and DNA methylation. A positive correlation has been reported between genes hypermethylated in cancer cells and genes bound by PRC2 subunits and the PRC2 histone mark H3K27me3 in embryonic stem (ES) and other cells [54–56]. Moreover, a large overlap between DNMT3B target genes in B cells and PRC1 and PRC2 target genes in ES cells or embryonic fibroblasts has been found in different cell types [57]. Studies with ES cells point to a transition from a PRC-bound state of certain genes in undifferentiated cells to a DNA methylated state in their differentiated progeny. Promoters which are occupied by H3K27me3 in ES cells are more likely to gain DNA methylation during differentiation [58]. Furthermore, loss of *Fbxl10* leads to a *de novo* methylation of CpG islands that are bound by PRC1 and PRC2 components, and this does not occur at CpG islands that are PRC-free [59], indicating that the presence of PRC might represent a prerequisite for this *de novo* DNA methylation. Interestingly, mixed states of PRC occupancy and DNA methylation have been found in somatic and cancer cells: loci with overlapping H3K27me3 and methylated DNA have been described [54, 60, 61] and genes that are enriched for H3K27me3 in normal cells can display H3K27me3 and DNA methylation or DNA methylation only in cancer cell lines of the same tissue [54, 62].

A possible explanation for these data is that silencing at certain loci might be induced by PRCs, followed by DNA methylation and subsequent loss of PRC binding at DNA-methylated regions. The circumstances and molecular mechanisms of such a scenario remain to be established. Potential hints at how PcG proteins are involved in DNA methylation are provided by studies showing that the PRC2 component EZH2, which catalyzes H3K27me3, can recruit DNMTs [63] and that BMI-1 forms a complex with DNMT1, DNMT1A and RING1B, and cooperates with DNMT1A [64]. In this context it is important to note that recent investigations have reported considerable *de novo* methylation activity of DNMT1 ([53] and references therein).

In the context of our study, both PRC1 and PRC2 components seem to be involved in the repression of *EphA7*. BMI-1 is part of both canonical and non-canonical PRC1, which can be found in the vicinity of the same gene [18] and the presence of H3K37me3- a histone mark known to be deposited by PRC2- preceded DNA methylation at the *EphA7* locus. An alternative explanation to a direct involvement of BMI-1 in mediating DNA methylation at this locus is that a decreased gene expression state *per se* may render the *EphA7* locus more prone to become DNA methylated and thus explains the late increase in DNA methylation upon *Bmi1* overexpression. In any case, slow increases in DNA methylation of BMI-1 target genes including *EphA7*might in turn make them more amenable to further silencing events, finally leading to a highly methylated state as found in tumor cells. Although our data suggest that a delayed DNA methylation process is initiated upon *Bmi1* overexpression, investigation of different stages of tumor development *in vivo* will be required to better understand the dynamics and mechanisms of *de novo* DNA methylation at the *EphA7* locus. Collectively, our study has revealed *EphA7* repression as a new mechanism by which BMI-1 controls cell proliferation in the postnatal LVW and provides novel molecular support for a connection between Polycomb group proteins and tumor suppressor gene silencing.

## MATERIALS AND METHODS

### Animals

*Bmi1* knockout mice (C57BL/6 background) were kindly provided by Maarten van Lohuizen [4]. *EphA7* knockout mice [35] were from Charles River/The Jackson Laboratory (*Epha7<tm1Ud>/J,* Bar Harbor, USA). This strain with a complex background was crossed onto a C57Bl/6 background. CD1 mice were from Charles River (Sulzfeld, Germany). The animals were housed in an animal facility and kept under standard conditions. All animal procedures were approved by a local ethical committee.

### Statistical analyses

Statistical analyses were performed with GraphPad Prism 6 (GraphPad Software, San Diego, USA) if not stated otherwise.

### Neurosphere culture and transduction

Neurospheres from postnatal and embryonic day E14.5 were isolated, cultured and transduced according to [65]. The pCMMP-IRES2-eGFP retroviral vector with mouse *Bmi1* cDNA, *Bmi1*-FLAG cDNA (C-terminal FLAG-tag) or without an insert (empty control) upstream of the IRES site was used [65]. GFP-positive cells were sorted using a FACSAriaIII (BD Biosciences, San Jose, USA).

### *In vitro* neurosphere assays

Neurosphere cells (NSPs) from 4 week-old CD1 mice were transduced with pCMMP-Bmi1, pCMMP-Bmi1-FLAG and empty vectors and plated in triplicates at clonal density (2,000 cells per well, i.e. 1 cell/μL or 1–2 cells/cm^2^) in standard 6-well plates (TPP, Trasadingen, Switzerland). At each passage (7–10 days after plating), NSPs were counted microscopically. After dissociation with Accutase and resuspension in NSP medium, viable cell numbers (identified by trypan blue dye exclusion; Sigma-Aldrich, St. Louis, USA) for each well were determined with a hemocytometer (Neubauer chamber; Roth, Karlsruhe, Germany).

### Microarray analysis

Microarray hybridizations were done as described in [65]. The data can be retrieved from the GEO database (accession number GSE70451). Normalized data were further analyzed with MaxDView (http://www.bioinf.manchester.ac.uk/microarray/maxd/maxdView/). A *t*-test was applied to compare *Bmi1*-overexpressing and empty vector control samples (*n* = 3 of each). Differential expression of genes was considered relevant in case of a p2value below 0.001 and a mean log difference of at least 1.

### ChIP

NSP cells from 4–6 week old CD1 mice transduced with pCMMP-Bmi1-FLAG and empty vectors were sorted by flow cytometry based on GFP and used for ChIP with an anti-FLAG antibody. For H3K27me ChIP, the same cellular material as for the DNA methylation analyses was used (transduced and sorted NSPs from 2–3 week old C57BL/6 mice). For details, see Supplementary Information Materials and Methods.

### Isolation, culture and transduction of B cells from mouse spleen

Spleen tissue from 6 week-old mice was homogenized in cell buffer (5% FBS and 2.5 mM EDTA in PBS), red blood cells were removed using NH_4_Cl and the resulting cell suspension was filtered (30 μm filter, BD Bioscience) and CD19-positive cells were isolated. Additional details, cell culture and transduction are described in Supplementary Information Materials and Methods.

### Cellularity of hematopoietic organs

Isolated thymus, spleen and bone marrow cells were counted with a hemocytometer (Neubauer chamber; Roth) (see also Supplementary Information Materials and Methods).

### qRT-PCR

Total RNA was isolated with the all prep RNA/DNA mini kit (Qiagen, Valencia, USA). RNA was converted to cDNA using M-MLV Reverse Transcriptase (Promega, Madison, USA) and random hexamers (Thermo Scientific, Waltham, USA). qPCR was performed with the Power SYBR green PCR master mix using a Step one Plus thermocycler (Applied Biosystems, Waltham, USA) with the following reaction conditions: 95°C for 10 min, 95°C for 30 s and 60°C for 1 min (except for EphA7-FL and EphA7-S, 63°C for 1:45 minute), steps 2–3 were repeated 45 times. Data were normalized to *Tbp*. Primer sequences are listed in Supplementary Information Materials and Methods.

### Immunostaining of tissue sections

Cryosections of brains from 6–7 week old mice were stained with rabbit anti-Ki67 (1:500, Novacastra laboratories, Wetzlar, Germany), goat anti-Dcx (1:100, Santa Cruz biotechnologies), mouse anti-Mash1 (BD Pharmingen). Secondary antibodies coupled with Cy3, Cy5 and Alexa488 (1:300, Jackson ImmunoResearch laboratories, West Grove, USA) were used (see also Supplementary Information Materials and Methods).

### Quantification of Ki67-positive cells

Coronal sections of anterior and posterior locations of the brain were analyzed. Sections were chosen based on visual hallmarks (anterior sections at around 1.0–0.9 mm from bregma, posterior sections about 0.02 mm from bregma (as defined in [66]).

Ki67-positive nuclei and DAPI-positive nuclei were counted around the dorsolateral corner of the LVW (100 μm medially and 250 μm ventrally). Only cells with a distinct colocalization of DAPI and Ki67 were considered Ki67-positive. For each brain, one representative section was analyzed and nuclei in the dorsolateral corner were counted in the two lateral ventricles present on each tissue section.

### DNA methylation analysis

Genomic DNA was isolated from NSPs (derived from 2–3 week old C57BL/6 mice) using the all prep DNA/RNA mini kit (Qiagen). Bisulfite conversion and purification was done with the Epimark Bisulfite Conversion Kit following the instructions of the manufacturer (New England Biolabs, Ipswich, USA). See Supplementary Information Materials and Methods. The frequency of methylated CpGs of each clone was calculated as ratio of the number of methylated CpGs/total number of CpGs.

### AZA treatment

NSPs kept in culture for 6 weeks after transduction with *Bmi1*-FLAG were plated at a density of 100,000 cells per well of a 6 well plate. Three days later, formed spheres were treated with 5 μM 5-Aza-2′-deoxycytidine (AZA, Sigma) for 48 h, with medium and drug renewal every 24 h. Cells were harvested, washed in cold PBS, and processed for RNA extraction.

### Calculation of Spearman's rank correlation coefficients

Raw Affymetrix gene expression data of human follicular lymphoma (Gene Expression Omnibus series GSE37088), human glioblastoma (GSE4290 and GSE4536), and human medulloblastoma GSE37382 and GSE37418) were analyzed using the R LIMMA package [67–69]. Expression estimates were calculated using RMA and normalized using quantile normalization. Spearman's rank correlation coefficients were calculated between human EPHA7 and 20 genes coding for components of mammalian PCR1 (RING1 [RING1A], RNF2 [RING1B], CBX2, CBX4, CBX6, CBX7, CBX8, PCGF1, PCGF2, PCGF3, BMI1 (PCGF4), PCGF5, PCGF6, PHC1, PHC2, PHC3) and PCR2 (EZH1, EZH2, EED, SUZ12) complexes using the R cor.test function [70, 71].

## SUPPLEMENTARY MATERIALS TABLES AND FIGURES






