# Supplementary material for: The putative tumor suppressor gene EphA7 is a novel BMI-1 target
Source: Oncotarget. 2016 Aug 13;7(36):58203–17. doi: 10.18632/oncotarget.11279 (PMC5295425; doi:10.18632/oncotarget.11279)
Supplement: Supplementary file 1 [file oncotarget-07-58203-s001.pdf]

The putative tumor suppressor gene *EphA7* is a novel BMI-1 target

Supplementary Materials

SUPPLEMENTARY MATERIALS AND METHODS

ChIP

To cross-link DNA and proteins, formaldehyde (Merck) was added to the culture medium at a final concentration of 1%. Cross-linking was allowed to proceed for 10 min at room temperature (RT) and stopped by addition of glycine (Scharlau, Barcelona, Spain) at a final concentration of 12.5 mM, followed by an additional incubation of 5 min. Cells were washed with ice cold PBS (Lonza) containing 2 mM $MgCl_2$ (OMNI Life Sciences, Billerica, USA) and resuspended in 1 mL lysis buffer (Dahl and Collas, 2008) with a protease inhibitor cocktail (Roche, Bromma, Sweden) and 1 mM PMSF (Roche). After an incubation of 20 min at RT, the cell suspension was transferred to 15 mL sample tubes (Greiner BioONE, Bad Haller, Austria) with 200 mg PBS-washed glass beads (Sigma-Aldrich) for disruption. The ice-cooled cells were sonicated four times for 30 sec with 1 min breaks in between at power level 4 and 50% duty cycle with a Branson 450 probe, yielding DNA fragments with a bulk size of 100–400 bp. The debris-cleared lysate was aliquoted and stored at −80°C.

Slurry of magnetic Dynabeads (Invitrogen) was mixed 1:2 with PBS-based blocking buffer (Lonza) containing 1 mg/mL Herring Sperm DNA (Promega) and 1 mg/mL BSA (molecular grade, Sigma-Aldrich) and incubated over night at 4°C on a rotator. For each reaction, beads contained in 100 µL of the slurry-blocking buffer mix were separated with a DynaMagTM magnet (Invitrogen) and incubated for 20 min at room temperature with monoclonal anti-FLAG-antibody M2 (5 µg, Sigma-Aldrich), anti H3K37me3 antibody (5 µg, Abcam) or IgG1-antibody (5 µg, Millipore, negative control) diluted in 200 µL binding & washing buffer (Invitrogen). For each immunoprecipitation, 100 µL cell lysate were diluted with 900 µL RIPA buffer (Dahl and Collas, 2008) containing PMSF and protease inhibitor and incubated with the prepared beads for 2 h at room temperature on a rotator. Beads were washed 3 times with washing buffer (Invitrogen) and then transferred to clean tubes. Cross-linking was reversed by resuspension of the beads in 250 µL digestion buffer containing 10 mM Tris (Scharlau), 10 mM EDTA (Merck) and 1% SDS (Calbiochem, Billerica, USA), addition of proteinase K (Fermentas, Waltham, USA) to a concentration of 50 µg/mL, and incubation for 2 h at 68°C on a shaker (800 rpm). 10 µL of raw lysate obtained after sonication were diluted in 240 µL digestion buffer and treated equally to the ChIP samples, serving as input control. Finally, the material was phenol-chloroform extracted and ethanol precipitated. DNA was resuspended in 50 µL of nuclease-free water (Ambion, Billerica, USA) and 2 µL were used as template for PCR. ChIP primers (listed below) to amplify mouse genomic sequences at BMI-1 target loci were designed as follows. Promoter sequences and transcription start sites (TSS) of putative direct BMI1 target genes were retrieved from the Transcriptional Regulatory Element Database (Michael Zhang Lab, Cold Spring Harbor Laboratory, USA; https://cb.utdallas.edu/cgi-bin/TRED/tred.cgi?process=home). Promoter regions were analyzed for CpG islands with EMBOSS Cpgplot (EMBL-EBI, Welcome Trust Genome Campus, UK).

ChIP primers were designed to bind to sequences within the respective promoter region, -750 upstream to 200 downstream of the TSS and to span identified CpG islands. Amplicons were not overlapping. ChIP primers for Trp53bp2 were designed to bind to sequences -1000 upstream to 1000 downstream of the start of exon 1. Ink4a primers were adapted from Matheu et al. (1) to cover the region -796 to -407 upstream the p16Ink4a ATG start codon. Amplified bands were quantified using ImageJ (https://imagej.nih.gov/ij/).

Amphotropic virus production

One day before transfection, 5 × 10^6 Phoenix packaging cells were plated per CellBIND 75 cm^2 flask (Corning Inc.). Cells were co-transfected with 10 µg of plasmid of interest (pCMMP-IRES2-Bmi1-eGFP, pCMMP-IRES2-*Bmi1*-FLAG-eGFP or pCMMP-IRES2-eGFP), 6 µg packaging plasmid pcDNA3-MLV and 1 µg envelope pMDG-VSVG by the calcium phosphate precipitation method. The transfection medium contained DMEM with GlutaMax (Life Technologies), 10% FBS, 1% sodium pyruvate (Life Technologies), 10 mM Hepes and 25 µM chloroquine. 8 h after transfection the medium was changed to remove chloroquine. 24 h after transfection normal Phoenix cell medium was added and viral supernatant harvested after 24 h and 48 h

post-medium change. Filtered supernatant was collected and incubated for 45 min on ice with Lenti-X ™ concentrator (Clontech, Mountain View, USA) at a ratio of 1:4. After a centrifugation for 45 min at 1500 × *g*, the pelleted viral particles were resuspended in appropriate medium.

Immunostaining of tissue sections

Tissue was fixed in 4% paraformaldehyde/PBS overnight at 4°C, transferred to 30% sucrose/PBS for cryoprotection overnight at 4°C, embedded with OCT Cryomount (Histolab, Gothenburg, Sweden), snap-frozen on dry ice and stored at −80°C. 10 µm coronal sections were cut on a Microm HM 560 cryostat, mounted on Superfrost Plus adhesion slides (Thermo Scientific) and stored at −80°C until further use. Sections were air-dried at RT for 15–20 min, incubated in 0.1% Triton X-100 (Sigma-Aldrich)/PBS for 5 min, washed 3 × 10 min in PBS and blocked in 5% donkey serum (DS) (Jackson ImmunoResearch laboratories)/DPBS with Ca and Mg (Lonza) for 1 h at RT. Primary antibodies were diluted in 5% DS/DPBS at 4°C overnight. Sections incubated without primary antibody served as negative control. Slides were then washed 3 × 10 minutes in PBS followed by a second blocking step in 5% DS/DPBS for 30 min at RT. Secondary antibodies were diluted in 5% DS/DPBS and incubated for 1 h at RT followed by 3 × 10 min washing steps in PBS, a 5 min incubation in water and 5 min incubation in 100% Ethanol at RT. Sections were then air-dried at RT for 25–30 min before they were mounted with Vectashield H-1000 (Vector, Olean, USA) containing 1 µg/mL DAPI (Serva, Oklahoma City, USA) and sealed with nail polish. Immunofluorescence was captured with an Axiovert 200 M microscope using AxioVision 4.6 software (Zeiss, Oberkochen, Germany).

Paraffin tissue sections

Brain tissue was fixed (4% formaldehyde/PBS overnight), dehydrated and paraffin-embedded in Entellan. Tissue sections were stained with Haematoxylin/Eosin. Images were taken with a ScanScope scanner (Leica) and analyzed using the ImageScope software.

DNA methylation analysis

A maximum of 2 µg of DNA per sample was bisulfite converted in a GeneAmp PCR system 9700 thermocycler (Applied Biosystem, Billerica, USA) using the following reaction conditions: 95°C for 5 min, 65°C for 30 min, 95°C for 5 min, 65°C for 60 min, 95°C for 5 min, 65°C for 90 min. Samples were then desulfonated and purified. 3–4 µL of the purified converted DNA was amplified using the Epimark hot start Taq DNA polymerase (New England Biolabs, Ipswich, USA). PCR conditions were: 95°C for 30 s , 95°C for 15 s , 52°C for 30 s , 68°C for 1 min and 68°C for 5 min , steps 2–4 were repeated 40 times. Primers are listed in Supplementary Materials and Methods. PCR fragments were cloned into the pCR4-TOPO cloning vector (Invitrogen), TOP10 one shot competent cells were transformed and grown on carbenicillin (100 µg/mL) agar plates. Clones were picked, checked by PCR, and sequenced by GATC Biotech.

Cellularity of hematopoietic organs

6–10 week-old animals were used. Thymi were dissected into cold R5 medium (RPMI medium, 5% FBS, 10 mM Hepes, 1% sodium pyruvate, 1% penicillin/streptomycin and 1% Partricin) and cut into small pieces (1 mm^3) using surgical blades. Tissue pieces were washed in 5 mL of R5 medium for 10–15 min under rotation at 4°C. After 5 min of sedimentation the supernatant was removed and centrifuged for 5 min at 300 × *g*. Sedimented tissue pieces were then removed and digested for 20 min at 37°C under 350 rpm shaking in 1 mL of digestion medium containing liberase TM (Roche) at 0.325 W/mL and 1/20 of DNAse I (Fermentas). Two rounds of digestion were performed. Cell supernatants were transferred into 10 mL MACS buffer (PBS, 2% FBS and 2 mM EDTA). All isolated cells were then centrifuged at 300 × *g*, resuspended in appropriate volume of MACS buffer at 4°C.

Spleens were dissected in cold R5 medium and mashed through a cell strainer with the piston of a 1 mL syringe. Bone marrow cells were harvested by crushing the femur, tibia, and iliac bone dissected from two legs with a mortar and pestle in cold PBS. The suspension was filtered with a 100 µm cell strainer. Isolated thymus, spleen and bone marrow cells were counted with a hemocytometer (Neubauer chamber; Roth).

Isolation, culture and transduction of B cells from mouse spleen

Cells were incubated with anti-CD19 biotin antibody (BD Pharmingen) at a concentration of 1/200 for 30 minutes at 4°C followed by an incubation with anti-biotin magnetic beads for 20 minutes at 4°C to specifically bind CD19+ B cells. CD19+ B cells were sorted with a magnetic sorting column mounted on a magnetic separator (MACS from Milteniyi Biotec, Bergisch Gladbach, Germany) and cultured in 6 well plates (TPP) at a density of 10^6 /mL in RPMI 1640 medium containing 15% FBS, 1x GlutaMax penicillin 100 U/mL and streptomycin 100 µg/mL, non-essential amino-acid, 50 µM β-mercaptoethanol, 20 µg/mL IL4 and 10 µg/mL of anti-CD40. 24 h later, isolated cells were transduced by the addition of 300 µL amphotropic viral supernatant and polybrene (final concentration 4 µg/mL, Sigma) to the medium. Medium was changed after 24 h and cells were kept in culture for 5 more days.

	Forward primer	Reverse primer
**ChIP primers**		
**Ink4a locus**		
Primer set 1	CAGATTGCCCTCCGATGACTTC	CCAAGCTGAGATCCCAACAACC
Primer set 2	GGTTCGACTCTAGGGTTGTTGG	TGGACCCGCACAGCAAAGAAGT
**EphA7 locus**		
Primer set 1	GTTGTTGTGAACTCAGGCAAAC	CGGACAGTGAAACTGAGCTATG
Primer set 2	TGCAGTAGCTAAAAGCCAAGTG	TTGCTCCACACTCCAATAATATC
**Ndn locus**		
Primer set 1	AACTCATCATCATCATAAGGTACAGC	GATTTCCTGGTCTCCTGTATGG
Primer set 2	GCCTAGTGGTACCCTCCCTTAG	CGCTCAGGTCCTTACTTTGTTC
**Rps6ka6 locus**		
Primer set 1	GTGTTGGGTGCACTTGTTTG	AGCGAGAGGCAGCACATAAT
Primer set 2	CCAGGCAGCTTCTTCTCG	CGGGCTGCAGACTTACAAC
**Trp53bp2 locus**		
Primer set 1	CCTAAACCCCTCATCAACTCAC	AAAAATTTAGGAACACCCTCCAC
Primer set 2	GGTGGAGCTTCGGGCTTC	CGGCTCTTGACCTTCAAAAC
**Tssc4 locus**		
Primer set 1	CTTGGCATGAAGAGACGTAGTG	CACTACGTCTCTTCATGCCAAG
**qPCR primers**		
**Tbp**	GGGAGAATCATGGACCAGAA	TTGCTGCTGCTGTCTTTGTT
**Bmi1-total**	CAAGATCACTGAGCTAAATCCCC	GGACAATACTTGCTGGTCTCC
**mEphA7-FL**	AGAGATGTTGCAGTGGCCATAA	TATCACAATCATGACAGGCTTCC
**mEphA7-S**	ATCCACCATACGTTGCATGC	GCACAACCGCTAGCTAATACA
**mEphA7-T1**	CATCATTGGAAGAAGGCACTG	TGCGGTTTATAAAACTGACAGG
**mEphA7-T2**	CATCATTGGAAGAAGGCACTG	TGTCTTTTCCATCCCGTCTC
**mp16**	GTGTGCATGACGTGCGGG	GCAGTTCGAATCTGCACCGTAG
**mp19**	GGCTAGAGAGGATCTTGAGAAGAGG	GCCCATCATCATCACCTGGTCCAGG
**Trp53bp2**	ATTTAGCCGAAGTGTGGTGTG	TGTTAACCCCATTCTCCTTCC
**Rps6ka6**	TCGTCAAACTGCACTATGCT	GGCCAATTCTGCAAGGTAGA
**Ndn**	GGCAGCTACAAGAAATGGTG	CACCCTGTCTAGCTCCTCT
**Bisulfite sequencing primers**		
**fragment b**	TTTTTTTTAATTGATATTATTGGAG	TATATACAAAACCAAACAACCAAAT
**fragment c**	ATTTGGTTGTTTGGTTTTGTATATA	AAACTCCACTCCCCAAATTTC
**fragment d**	GAAATTTGGGGAGTGGAGTTT	CCTATAATCAAAAAAAACCTC

REFERENCES

1. Matheu A, Klatt P, Serrano M. Regulation of the INK4a/ARF locus by histone deacetylase inhibitors. J Biol Chem. 280:42433–41.

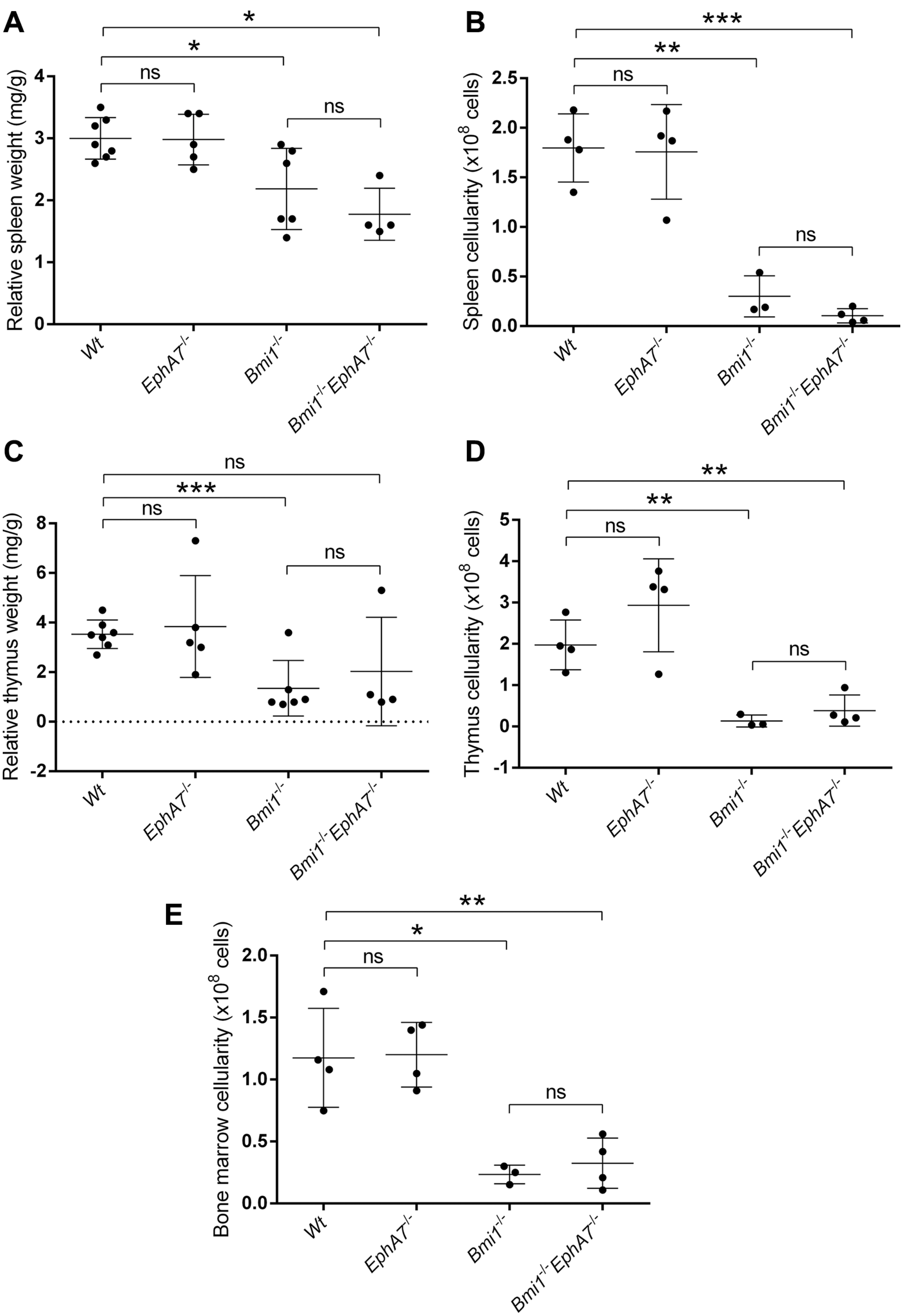

Supplementary Figure S1: Deletion of *EphA7* does not rescue hematological defects in postnatal *Bmi1*$^{-/-}$ mice. Scatter dot plots depicting mean values with standard deviation of relative spleen and thymus weights (organ weight in mg/animal weight in g) and spleen, thymus and bone marrow cellularity. *p*-values of unpaired *t*-tests: *$p \leq 0.05$, **$p \leq 0.01$, ***$p \leq 0.001$, ns: not significant ($p > 0.05$).

Supplementary Information File S1: Genes differentially expressed between *Bmi1* overexpressing and empty control neurosphere cells. See Supplementary Information_File_S1

Supplementary Information File S2: Correlation analyses of human EPHA7 expression levels and genes encoding PRC1 and PRC2 components. See Supplementary Information_File_S2
